# Speculations on the Facts Presented in the Analytical Report on Mesmeric Somniloquism

**Published:** 1844-04

**Authors:** Daniel Drake

**Affiliations:** Louisville


### WESTERN JOURNAL

OF

### MEDICINE AND SURGERY.

---

APRIL, 1844.

---

### ART. I.—*Speculations on the Facts presented in the Analytical Report on Mesmeric Somniloquism, published in the last number of this Journal.* By DANIEL DRAKE, M.D.

The estimate of the phenomena of Mesmerism, by different minds, presents us with examples of the easiest credulity, and the most obstinate unbelief, not less than a reasonable scepticism. This ultraism has arisen from want of analysis. Among the phenomena there are some, which have their prototype in nature; others which have not. To indulge a spirit of infidelity in regard to the former, is quite as absurd, as to cherish credulity in reference to the latter; and still, many persons have grouped the whole, and affirmed or denied their truth

*en masse.* A rational scepticism will not admit the reality of the lowest, without evidence; nor refuse to grant that the highest and most startling *may* be true. It will, however, demand for the latter an amount of proof far greater than for the former—an amount corresponding with their wider departure from the observed and ascertained phenomena of nature. Of the two classes of phenomena, presented by mesmeric somniloquism, one bears a close resemblance to those which characterize natural sleep-talking—the other are not yet known to belong to that state. Now the degree of proof on which we may admit the former, is much less than would justify our admission of the latter. Let us inquire separately into their claims on our belief.

The lowest degree of natural sleep-walking and sleep-talking, is that in which the individual while in ordinary sleep, may strike at an imaginary object, or sit up in bed, or pronounce a sentence, as asking or answering a question—uttering an exclamation, or giving a command. In a higher degree, he will rise and walk about, or, continuing in bed, carry on a conversation with an imaginary person, or sing or declaim. When up, he will wander among objects in the dark without encountering them, even when blindfolded; and should he undertake to perform a work which is generally directed by the sense of sight, he appears to be capable of going on with it, while screens impenetrable to light, are interposed between his eye and hand. At other times he uses light, or *seems* to use it, and has been known, when his candle was snuffed out, to re-light it, and then proceed with his manipulation. Sometimes he stumbles over objects to a degree which indicates that he does not recognise them, and occasionally mistakes one for another. In this state, whether silent or talkative, he is generally inattentive to what is said to him, and even to the loudest noises, as he is insensible, in a great degree, to bright lights, to strong odors, and to impressions, whether painful or pleasurable, on his nerves of feeling. But, every now and then, he will unexpectedly address himself to an attendant, whom he had immediately before refused to recognize; and in other instances, after many questions had been asked without answers being returned, he has made a distinct reply. Whether he himself had put the question or made the reply, the individual thus recognized may generally, thenceforth, maintain intercourse with him, and more or less control the current of his thoughts and actions. This condition is often brought on by a slight degree of bodily indisposition, by a supper of indigestible food, by a previous highly or painfully excited state of feeling, or by remarks and questions patiently whispered in the ear. Occasionally we find it to affect several members of the same family. From this condition the patient may be aroused, but often returns to his bed, and the somnambulic gives place to natural sleep. Whether he awake from one or the other, he has, in general, no recollection of any thing but a dream. Hence somnambulism and somniloquism, have been defined to be dramatised dreams. The dream is often recollected, but in many instances, as in common dreams, it cannot be recalled. It is probable, that in these dreams, although the imagination of course is active, as in all cases of dreaming, the comparing or reasoning powers are more awake than in ordinary dreams, which are too inconsistent and disjointed to prompt to any kind of action; in proof of which, I may refer to the practical ability displayed by many somnambulists, who pass uninjured through difficult and perilous places, though, sometimes, they destroy themselves by deliberate acts, unaware of the danger before them; but the activity of the reasoning faculties is occasionally evinced, in a still more conclusive way, by their literary compositions, and the mathematical solutions which they have been known to work out. Such dreams bear much resemblance to monomania, or derangement on one subject; while ordinary dreaming has, in its incoherent, grotesque and ever-varying creations of fancy, a close resemblance to general mania, in which the mind does not associate well, is not fixed on any single group of ideas long enough to compare them; and of course the body, although agitated, is not prompted to any definite or sustained mode of action. In the absence of all fixed purpose, there is an equal absence of defined and efficient execution.

There are two modifications of somniloquism and somnambulism which should be recognized in this inquiry. The first is the reverie, which sometimes alternates with convulsions, in which the individual displays a strong current of connected thoughts with appropriate feelings, accompanied with suitable action; but is wholly inattentive to all surrounding objects or persons; except when they, or what they say, can be incorporated with the catention of ideas. This state of mind is generally of short duration. The second may be called a protracted reverie, or prolonged somnambulism; continuing for days, and even weeks, during which the individual will act and converse with those around him, in an altered manner, and not in full sympathy with them. In coming out of this condition, the mind takes up the ideas on which it happened to be occupied at the access of the paroxysm; and is unconscious of its having existed; indeed, may remember no part of it; but upon the return of the fit will recollect the whole.

The principal characteristics then, of ordinary somnambulism and somniloquism, including, in part at least, the curious varieties just mentioned, are the following:

1. They occur chiefly in young persons of both sexes, in those of a delicate nervous system, and in connexion with bad health.

2. In some cases, the sense of sight seems to be greatly increased in acuteness, or that of feeling, or the instinct of the individual in some mysterious way, is substituted for it.

3. There is great abstraction. The attention of the person is entirely concentrated on the train of thoughts which is passing through his mind; and he is, consequently, insensible to what is around him, and even to violence on his body. But if, by chance or perseverance, his attention should be gained, he may in general be guided both in his thoughts and actions. His state of mind may be modified without his being awakened.

4. There is a spontaneous, inherent activity of imagination, which excites into action the muscles of locomotion and speech.

Let us now compare mesmeric, with natural somnambulism and somniloquism.

1. It is chiefly producible in children and young persons of both sexes; in individuals of frail and susceptible nervous systems; and in natural sleep-walkers, or members of families in which somnambulism prevails.

2. Of all the alleged phenomena of this state, none have excited more wonder, than those connected with the sense of sight; which has been said to be greatly increased in acuteness, and even transferred from the optic to other nerves. This is the *clairvoyance* of writers on mesmerism.

3. In this condition, the abstraction of mind is so great, that the individual is inattentive to impressions which in the waking state would give acute pain; and cannot be spoken with, except by the mesmeriser, who had her attention from the beginning, or by persons introduced by him.

These analogies between ordinary and mesmeric somniloquism, if not overcome by a greater number of differences, must lead to the conclusion, that they are but varieties of the same curious condition of the nervous system. Now what are the contrarieties? They seem to me to be the two following: 1st. The mesmeric state is more cataleptic—attended with less locomotion, and displays much less of a somnambulic character. 2d. It is attended with less talking, the individual seldom speaking except when spoken to, then generally answering in a single sentence, and relapsing into silence. From these two facts we may conclude, that the mind is inactive, that the animating dream is wanting, and, of course, there is absence of spontaneous walking and talking. If locomotion and loquacity were added, by an active instead of a passive state of the imagination, the two conditions—ordinary and mesmeric—would appear to be identical.

There is then no credulity in admitting the reality of mesmeric somniloquism; and although, no doubt, it is often simulated for gain, I am disposed to regard it as a fact, and reason upon it accordingly.

It is affirmed, however, that a peculiar sympathy of both body and mind exists on the part of the mesmeric somniloquist with the mesmerizer and those introduced, or as the technical phrase is, put in communication. But this sympathy is not reciprocal. It is confined to the somniloquist, who, it is asserted, can be made to experience the same feelings of both body and mind, and entertain the same thoughts, as the person in conversation with her, and this in some unknown manner by some occult influence, altogether independent of the ordinary means of intercourse by the senses. Let it here be particularly noted, that it is not a stimulation of the body or mind of the somniloquist into increased activity, her own sensations and thoughts being the objects of her consciousness; but an actual infusion of the feelings and thoughts of the person in communication, at the expense of those belonging to the somniloquist, and that, too, to such a degree, that if she had pleasurable sensations of the body before, she would, in the midst of them suffer pain, if his body were wounded; and although she might retain her own consciousness so far as to understand and answer questions, put to her through the medium of the ear; still that her predominant ideas are those impressed on her mind, which arise simultaneously with their origin in the mind of the person who is in communication. This is the mysterious and incomprehensible mental state of the mesmeric somniloquist, and to the ascertainment of its reality, the experiments of the association were directed.

Before proceeding to speculate upon them, we must refer to what seems to us almost an insuperable difficulty.

The proposition is, that the person in communication raises in the somniloquist a state of mind identical with his own: if so, how can conversation be maintained? Will he not supply the answers as well as the questions? And as long as he remains in communication, how can the somniloquist have any thoughts of her own? Or how can she have them at one moment, and not at another when the stream of influence is perpetual, seeing that it is not under the control of the will, and that the person in communication thinks incessantly? In ordinary circumstances when a question is asked, the person to whom it is put, is left to frame the answer according to the laws of his own mind, and the kind and amount of his own knowledge; but in the case we are considering, nothing is spoken, nor is there any effort made by the person in communication, except that of thinking with energy. When he has done this for a short time, he wishes to know its effect, and then frames and puts the question; but in doing this, his state of mind necessarily changes, and he becomes attentive to the expected answer. Now how does it happen, that this new state of mind is not impressed on that of the somniloquist, like that which immediately preceded it? But if impressed, it must bring hers into the same condition with his own, that is, waiting for a reply, and of course she could not make it: if not impressed, it is certainly an argument against the infusion into her mind of what he first thought over. The alleged ability of the somniloquist to give an account of her consciousness, while the person remains in communication, during which, according to the terms of the case, she has *his* thoughts, is, then, a paradox, and seems to be an absurdity.

I will not, however, dwell on this difficulty, but proceed to state and discuss the subject of intellectual sympathy in as fair and candid a manner as possible.

The proposition is, that when a person is put into communication with one who is in mesmeric somniloquism, a secret agent or influence passes from him, and raises in her perceptions and thoughts identical with his own; the evidence of which is furnished by the answers which she gives to the questions, which are put to her by the mouth through the ear. The point I mean to discuss is, whether, in reference to the report I am reviewing, it is necessary to adopt the theory of a mysterious agency, to account for the true or conformable answers it contains. I shall *assume*, not affirm, that it is not, and proceed to suggest how most of her answers might have been brought out, according to the established laws of the human mind.

It is an undeniable fact, that a mesmeric somniloquist is not asleep, nor in delirium, insanity or idiotism, but in an extremely passive and quiescent state of mind—not so sluggish and insensible as not to comprehend a question, but too torpid to put forth mental manifestations, without its stimulus. And herein lies the most obvious difference between her intellectual condition and that of a natural somniloquist, who is made to walk and talk, by the quickening impulse on his organs of locomotion and speech, of a dream or a reverie, the essence of which is an excited imagination. What such an one, (the natural somniloquist), sees, is of course the creation of his own mind; what he does is prompted by his dream. The current of his thoughts is strong—too strong, in most cases, to be interrupted, and the individual who happens to get into communication with him, will, in many cases, be compelled to go with the current, or part company; sometimes, however, he may get the mastery, and by his questions and remarks turn the stream of thought into other channels. The mind of the mesmeric somniloquist, like stagnant water, is without this current, but is capable of being excited by external influences, as the pool may be agitated by mechanical force. According to the nature, direction and mode of the action of this force, the undulations and currents established by it will vary; and in the same way, when the external influences, the remarks, and interrogations, which are brought to bear upon the mesmeric somniloquist may vary in their substance or manner, they will raise in her a variety of mental conceptions: stir up her imagination to various creations. Of these creations she is immediately conscious, and her replies express that consciousness. Thus the question itself is what arouses her imagination, and the answer announces, not what existed in her mind by secret infusion previously to his putting the question, but what was created between the time of hearing the interrogatory, and sending forth the reply. Hence the necessity of a lapse of time between the question and the answer. In the beginning of a conversation this is sometimes to be counted by minutes; but after the image of a particular object is once formed in the mind, the answers concerning its properties and parts, are obtained in more rapid succession; because when the imagination has once decided on the object, the various characteristics of it may be created instantaneously, as in the waking state, and still more in dreams. The chief difficulty lies in getting it to decide. Sometimes, however, this may be instantaneous, because what is said may suggest some object or several, one of which, according to the laws of suggestion will, *instanter*, be adopted. Thus, to come to the facts of the Report, when Mr. C. D. asked, what have I in my hand? a horse, a man, a landscape, a boat, and all other objects not capable of being held in the hand, would be instantly rejected, and the imagination would only have to select out of those which could be grasped. In doing this it would of course choose one that was familiar, because familiar ideas would first come up. Hence, although the somniloquist might have heard of, or occasionally seen, a Hindoo idol, a pine-apple, a pocket-compass, or a silver lancet-case; neither of these objects, under the laws of mental association, could present itself to the exclusion of the objects with which her mind was previously familiar; and hence she answered "a book," and, under a series of questions which did not deny the truth of her answer, described it as such; although what he held and saw, *was* a silver lancet-case. Again. When Mr. I. J., while looking at a book, as a first question, asked her what she saw, she answered "a building;" but when he asked whether he held anything in his hand? she answered something white; and under a series of questions, concerning its properties, at last had the true image raised in her mind, and it seemed to her like a book. Further, when Mr. K. L. asked her to visit England with him, it was not at all probable that after having, in imagination, reached that country, she would on being questioned see a watch in his hand, or the interior of the Mammoth cave, or a group of Indians, but some object, of which she had heard or read, as attracting the attention of travelers, in that country, and, almost, as a matter of course, her imagination presented her with the image of an old stone church, of a peculiar kind. Further still. When Mr. G. H., without having directed her attention to any foreign place, proposed to take a walk and see fine things, her imagination would not present the scenery or objects of distant lands, nor the people, and drays of the streets of Louisville, but some object belonging to the class of pleasant sights, and it fixed on a large building; and when he asked, what of the top? her imagination was so directed, as to present a spire, the object present in his mind, and quite familiar to her own from being seen every day. Again. When he asked her to cross the mountains with him, it would at once raise in her the idea of objects on that side; of which the most impressive are in its sea-ports, and instead of seeing cotton fields, the ruins of Panama, or a book, she would of necessity, the laws of mental suggestion being in force, see something which belongs to the region where in imagination she had gone; and that something was a building, not very high nor very low, with columns; although the Washington monument, at Baltimore, was in his mind; and when he asked what was to be seen from the top of it, her fancy would not be likely to picture a cat or a snuff box, or a painting, but to create, as it did, a panorama of hills, water and houses. When Mr. C. D. asked her what she saw, she promptly answered a large house, which was correct, and when he inquired, what peculiarity? she answered columns, which was likewise correct. What, at that moment, determined her mind to fix on a building, cannot be known, any more than we can know what suggested to her the same object, while Mr. I. J. was looking at a Hebrew bible, and Mr. G. H. thinking of a human skull. Such answers as the latter relieve us from the necessity of concluding, that Mr. C. D. had sympathetically impressed her with the image of a building; from which we are still further relieved by the fact, that her imagination immediately entered the house which it had created, and consistently presented her with rooms, and persons standing, walking and sitting; though Mr. C. D., mean while, had, in imagination, descended into the Mammoth cave, and was threading its strange labyrinths. Had he, in thought, gone into the house instead of the cave, her answers would have had such conformity to the topic in his mind, as to favor the idea of a transfusion of ideas, while it would have been but an accidental coincidence. As long as the gentlemen in communication kept themselves in the midst of common scenes and familiar objects, these coincidences would, of course, be frequent; but when they recollected others, of which she was ignorant, her imagination created little or nothing that was applicable to them. Thus, when Mr. C. D. as just mentioned, reproduced in his mind the interior of the cave, as Mr. M. N. before him had done; when Mr. E. F. recollected a steam-boat scene on the Northern Lakes, and a walk on Santa Rosa Island in the Gulf of Mexico; when Mr. O. P. took her into a Rolling mill; when three different gentlemen conducted her to the anatomical lecture-room of the Institute; and when Mr. C. D. ascended the Ohio river in a skiff, on a fowling voyage, she saw nothing that indicated the slightest sympathy with them. The state of mind in which her answers were framed, may be compared to that of young persons in the play—*What are my thoughts like?* The young lady to whom the question is put, if she know nothing of the habits of thought, pursuits or character of him who puts it, exercises her imagination in framing the answer, entirely on her own knowledge, and often finds, as all who have engaged in this intellectual pastime know, not a little difficulty in settling upon any thing. If the gentleman were, at the moment, thinking upon some strange or distant object, of which she knew nothing, her answer would have no relation to it, but if he kept himself within the range of those scenes and objects with which she was intimate, and out of which she must of necessity frame her reply, it would often happen, that she would seem to have apprehended his thoughts.

It is easy on this theory to explain why the somniloquist so often answered, "persons," and "houses," and "water," when they really made a part of what was recollected by those in communication with her. The first she announced, correctly or incorrectly, about 30 times, and they were embraced about as often in the topics thought over by the gentlemen. The second occurred 24 or 25 times, and were presented about as frequently by those who were in communication with her. Of the third nearly the same might be related. Persons and houses, or buildings and water were, indeed, more common in the subjects chosen for our trials with her, than any others. Now, why did this happen? Undoubtedly, because those objects are so common—because we are social and domestic beings; and in setting our imaginations to selecting or inventing topics, they would naturally, that is according to the laws by which our thoughts are associated, fix on scenes and objects of a familiar kind; and the same would be true of her; for if her ideas had not been governed by the same laws of suggestion as ours, we could not have held conversation with her. Such being the fact, when her drowsy attention was awakened by the question—*what do you see?* her imagination if *it* framed the answer, would of necessity, and for the same reason as in our waking hours, present her with those common objects. And not *only* for the same, but the additional reason, that she, being a woman, had been all her life essentially domestic—residing in the midst of houses and people. It is worthy of remark, that in many of the instances in which the objects just named, made a part of the scenes presented by us, they were not those on which our minds dwelt with *most* intensity, but merely constituted the necessary accompaniments of the scene, or were the actors in some operation which we thought over; but with her, instead of being subordinates, they were principals. Why, however, if her mind had sympathized with ours, should she not have passed *by* the houses and spectators, and spoken of the curious objects and doings, which occupied *our* imaginations? On the theory of her answers being the product of her own imagination, thrown into activity by the questions, this paramount reference to houses and persons is fully explained; while it is utterly incompatible with the hypothesis of transfusion. On the same theory, had she been the somniloquent daughter of an Arab Sheik, she would have seen tents, and horses, and camels, and long beards, for *they* would have been her familiars. Now, if we reduce the number of conformable answers, in which persons, and groups, and water were the objects, and other things, equally or more impressive contained in the groups, were omitted, to their proper level, that is, until they shall bear a due proportion to the rest of her conformable replies, a number of the most correct will be set aside. But on what principle should this be done? I answer, that whenever it is obvious, that other things or operations, must have been equally or more vividly present in the mind of the gentleman, than persons, and houses, and water, but *she* did not see them, her mentioning the latter is no evidence of mental sympathy or transfusion, for if the fainter ideas of the gentleman could be impressed on her mind, his vivid thoughts would much more certainly impress themselves. There seems to me to be no escaping from this conclusion. A few examples may make the argument plainer. We have already seen, that when two gentlemen made their ideal visits with her to the Mammoth cave, she saw houses and people, but she saw nothing in the cave, although its impressive effect on their own minds, at the times of their actual visits, must have enabled them to recall its grotesque alcoves, snow-white stalactites and brilliant torch lights, far more vividly than the boarding-house at the entrance; which interested them no further than as a place of repose and preparation for the thrilling scenes of their subterranean visits. Again: At Colonel Thompson's she saw rooms and people, but why did she not more clearly perceive the blooming green-house, so strikingly contrasted with the desolate winter scenery of the surrounding woods and fields? Again: At the bull-baiting in England, she saw people, but not the object that brought them together, until she was asked if she saw any beasts. Further, when three gentlemen at different times conducted her to the amphitheatre of the Institute, she saw a room and persons, but did she see the striking objects peculiar to the place? Certainly not; and still it was to show her *them* that she was taken there. When two gentlemen thought over the scenes in the public lecture room, where she had been exhibited, she saw a house and people, but perceived nothing of the operations which were recalled by them. Finally, when conducted to the church in Cincinnati, she spoke of a house and people, but saw nothing of the picturesque and impressive ceremony, which made the essential part of the scene. These cases might be greatly multiplied, and it seems to me that a sound philosophical logic rejects them all, as instances of the transfusion of ideas into her mind. If not, it follows, that she sympathizes more with the weaker than the stronger recollections of the person in communication, which is equivalent to saying that the feeble is more powerful than the strong—the less superior to the greater—a part larger than the whole.

According to the views which I have presented, the questions put to her, both occasion the creations of her own imagination, which are what she sees, and call forth an expression of them in her answers. I wish the reader not to lose sight of this proposition, but, remembering it, to proceed with the me still further, in the inquiry how far, as a theory, it will explain the facts before us.

All our intercourse with her convinced us, that she was in a drowsy, inert, and feeble state of mind, especially in its reasoning faculties, or else affected it. We have assumed the former. Now, to such a mind, the stimulus of a question is like a galvanic shock to one apparently dead, which excites a momentary, convulsive and irregular action of the muscles. The weaker and more quiescent the mind, the greater is the power of another mind over it; when exerted in the common manner of remark, command or interrogation—in other words, the more will it be directed and moulded:—The less its inherent energy, the more will it be indebted to external influences for its activity; and the more will that activity take its coloring, form and character from those influences. That *her* mind was susceptible of being thus influenced, is evident from many parts of the record, and is a fact admitted by all who have conversed with her. But I need not rely exclusively on the late experiments for proof, that the question, and even the manner of expressing it, as well as the previous remark, will influence the reply; for in the Western Journal for June, 1842, there is a report of a series of experiments on corporeal sympathy, made in Cincinnati, in the presence of many respectable witnesses, which conclusively shows, that by varying the questions, or introducing them with different prefatory remarks, the same water and the same bread and meat, were made to have, respectively, many different tastes, and to be a variety of things, according to the varying creations of the imagination of the somniloquist. Such results would seem to show conclusively, that the answers do not express the ideas of the person in communication, secretly imparted, but the *effects* of *his* conversation and questions on *her own* imagination. The things seen by her are inherent—not transfused. She is *acted on by*, but does not *sympathize with* her companion. She sees what she would not see but for his influence; but she does not see what he sees, except by accidental coincidence, or except his questions or remarks be so framed, as to cause her imagination to bring forth the likeness of what is in his mind. That this may be done in the waking state, any two individuals may satisfy themselves by experiments of the kind made on her; but if practicable in that state, it may, *a fortiori*, be done in the mesmeric slumber, when the judgment is inert, the will nearly annihilated, and the external senses inattentive; when she is acted on, it is said, by no voice but that of her companion, and, as far as observation instructs us, has no mental activity but what his voice inspires.

If this theory be correct, it follows as a consequence, that a mesmerised somniloquist, may, by a protracted and skilful course of interrogation, be made to see almost any object which the person in conversation may choose to select; and this we may presume is the key to the striking results which are often obtained in public exhibitions. Those who engage in these exercises, although unskilful in conducting a course of interrogation, designed to reveal the true character of the mental state of the somniloquist, are instinctively qualified to bring out striking results. They feel, that courtesy and fairness require, that they should, by their questions, not mislead the somniloquist, but rather afford her some general intimation of the locality or nature of the scene or object they have in view; which intimation being given, and followed by questions on the details, they set her imagination to work within the prescribed field, and at last are astonished at the creations, which, under their own dictation, it presents to them—creations that sometimes even go in advance of their own minds, which come in turn to be led by the somniloquist. An examiner of this kind, is in the condition of a man, who, in a dead calm, might approach an Æolian harp, to hear its whispering melody, and mistake the music struck out by his own breathings on its strings, for that of breezes, too gentle, as he would believe, to be perceived.

A comparison of the instances in which the same scene or object was, more than once thought over by some of us, while in communication with the somniloquist, gives results at utter variance with the hypothesis of transfusion, but in harmony with that of interrogation. It will be seen by reference to the Report that Nos. 2 and 9, Nos. 4 and 21, Nos. 6 and 36, Nos. 12, 13 and 14, Nos. 15 and 16, Nos. 19, 40 and 50, Nos. 37 and 45, Nos. 38 and 46, and Nos. 39 and 47, were respectively the same topics; and on the hypothesis of mental sympathy, her account of each, as given to different gentlemen, or to the same at different times, should have been substantially the same. An examination of her answers under those different heads shows nothing of the kind. On the contrary, it is obvious at the first glance, that in all these cases, her own imagination created her answers. This may be affirmed with the more confidence from the fact, that these were not trials to see if she would answer in the same way, when questioned by different persons concerning the same scenes, for the fact of gentlemen having selected the same, was not known even to themselves, till the experiments were completed. Of the whole, certainly none are more important than these, which are not unlike the cross examination of a witness in court, or the *experimentum crucis* of the philosophers. They rest as a dead weight on the hypothesis of mental sympathy, but give a living support to the theory of interrogative stimulation.

There are other ascertained and admitted facts, which have the same bearing, two of which occur to me at this moment. One is, that the alleged mental transfusion is interrupted when the visiter, in communication, puts his question; but this could not be the case if the question did not modify the state of her mind; the other is, that by cross questioning, her mind may be misled and confused, so that she loses the whole or a part of what has been said to be transfused, and, therefore, gives vague or erroneous answers. Now what is all this, but to elevate interrogation over transfusion, as a means of securing her attention and creating new states of mind? But if interrogation have in it this paramount power, why not deduce from it all the results, seeing that it *may* be so conducted as to direct the attention and guide the imagination, even in the waking state? A cause adequate to the production of a phenomenon being discovered, it is not only su perfluous but unphilosophical to look for another. It is even absurd, if that assigned be a reality, and one which the mind can appreciate, to propose another that is at once hypothetical and incomprehensible. But I must not dismiss the subject of interrogation here. It is asserted by observers of the phenomena of mesmeric somniloquism, that persons in communication, who are dull and unimpressive in their mode of interrogating, do not obtain as lively and proper answers, as those who put their questions with animation, and occasionally enliven the somniloquist with sprightly or mirthful sallies. Now from the phenomena of natural somniloquism, as well as from reasoning on the subject, I would say, that in proportion as these measures might awaken her imagination and give it impulse, the reception of transfused ideas would be resisted, and the phenomena depending on mental sympathy with her companion, diminished. But, on the theory we are supporting—that all she gives out is of home manufacture—the more her imagination might be aroused by conversation, the brighter and better would be its creations, and the more definite her replies. Moreover, it does not follow, that he who puts his questions in a feeble and frigid manner, has inert feelings or a dull imagination—but rather the reverse; as intense effort in the reproduction of a scene is unfavorable to earnestness and playfulness of expression. To say, indeed, that lively questioning is necessary to procure lively responses, seems an abandonment of the theory of transfusion, which should be great in proportion to the intensity of thought—not the energy of speech; but on the theory we advocate, intensity of thought is unnecessary—sprightliness of interrogation everything.

It may be said, that in several instances during our recent experiments, and at other times and with other somniloquists, there have been prompt answers as to individual objects, which were correct. This is true, but the conclusion from it is, logically, false; for first, the objects concerning which she answered correctly, without a course of previous questioning, were scarcely any others, than "buildings," "people," "water," and "something moving;" which she was perpetually announcing; and second, in an equal number of times, her first answers were incorrect. It may be said, however, that the latter were mistakes. But does not their occurrence prove, that answers *were* given by her in opposition to the state of mind of the gentleman in communication with her? and is not this at variance with the hypothesis of transfusion? She could not answer a "book," a "building," "people," or "water," unless the idea of it were present in her mind. But if it were not in the mind of her companion, at the time, it could not have been transfused, but must have been created by her own imagination. Here then we see that in the very midst of the alleged transfusion of the ideas relating to one object, her own imagination created the image of another.

Now it is according to all human experience, that *some* of these creations should by accident be true to what was in the mind of her companion. If while writing this paragraph, I should ask a blind man which I was using, the quill of a goose, a swan, a turkey, a vulture or an eagle, he might anwer the first and be correct, but would that prove that he saw it? Certainly not. He was limited in his choice to the five, and, therefore, the chances against a successful answer, were not indefinitely great. But his knowledge of the common use of the first, or its being the first named, might in his judgment have rendered it probable that I was writing with it. The somniloquist might not exercise this judgment, when asked what I held in my hand, but, in framing an answer, she must of necessity fix on some object of a familiar kind, commonly held in the hand, which might or might not be the true one. If the blind man had known, that I made an equal use of the five kinds of quills, his first thought would probably have been of the one which had been most used by himself; and thus, the somniloquist, when asked "what have I in my hand?" necessarily thinks first (as I have already shown) of some familiar object: and having, in her torpid condition, no power of the understanding to inquire into the probability of the answer, gives it out in full confidence of its being right, although it *may* be either right or wrong. She believes it correct, because there is nothing in her somnolent judgment to oppose her conclusion. In this respect her state of mind is identical with that of a madman, who has full faith in the creations of his distempered imagination, because he is incapable of comparing them with anything else —cannot subject them to the test of his senses or his reason. Yet his creations of fancy might be according to the reality, as, for example, that his house was surrounded by robbers, when it really was; but no sound mind would believe from the coincidence, that he had been mysteriously informed of the fact, by sympathy with another mind. I cannot but believe, then, that logic supplies a lever with which to overturn that superstructure of faith in transfusion, which has been erected on the occasional coincidences which our report furnishes. In the nature of things they must have occurred.

If mental sympathy really existed in the mesmeric state, we ought to hear of exercises of the thinking faculties, by some of these somniloquists, but nothing of the kind has, I believe, been exhibited. All the phenomena depend on the imagination. We hear little or nothing of mathematical solutions, philosophical inductions, or geographical relations. Contrarieties and discrepancies of all kinds are entirely overlooked. Harmonies are neither produced, sought after nor missed by the somniloquist. There is no abstraction, no generalization; and the whole mental performance is but the creation and exhibition of a series of phantasmagorial pictures, or rather of their imperfect, shapeless and uncombinable elements.

And this leads me to speak of the relations of the imagination with the sense of vision. In our experiments, no allusion was made to sensations in the organs of touch, taste or smell, and but three or four times to those of hearing, and then, generally, in consequence of a leading question; and with the simple enunciation of sound, without applying to it any epithets expressive of its melody or harmony. In fact, almost every idea awakened in her was visual, which is precisely what would happen if her *own* imagination supplied the objects of her consciousness; while if the feelings and thoughts of the person in communication were transfused, I can see no possible reason, why manifestations should not have been made of the perceptions received through the other senses. It is a well known fact, that the sense of vision supplies the imagination with its principal materials. Hence all poetry, except the didactic and pathetic, abounds in visual ideas, which, moreover, enter largely into the tropes and metaphors of the kinds I have mentioned as partial exceptions. The vividness of the ideas obtained through the eye, is, moreover, very great, and so attractive to our attention over those of the other external senses, that we speak of seeing, as synonimous with examination by means of all the other sentient organs. This popular phraseology shows the experience of the world, in regard to the superiority of this sense. The sensations of the eye, moreover, are not only impressive at the moment, but abide with us; and after some time, are almost the only ones we can revive. Thus, many years after having discovered a new plant, we are able to recall the appearance of its flowers, and that of the wild scenery where it grew, much more vividly than its odour, the feel of its leaves, the taste of its bark, or the song of the birds in the surrounding trees. Indeed, until we recall the visible appearance of an object, it does not seem to be present in the mind; but when once reinstated, as an object of sight, its qualities and properties, as ascertained by our other senses, are gradually, though much more faintly reproduced. Thus it is, that the magazines of the mind are almost filled with visual perceptions, and the imagination is not only the keeper of the key, but the artist which weaves them into tissues. Almost all its creations, therefore, necessarily address themselves to the "mind's eye," not to its ear. In general these reproductions cannot be mistaken for present impressions on the nerves of vision, for two reasons; 1st. The exercise of the other senses assures the individual, that what he sees is external to him; 2d. In the absence of the opportunity of thus exercising his senses, he can reason on the visual presentation, and decide upon its character. But when neither of these correctives, or opposing influences operates, it becomes *inevitable*, that the image or picture created by the imagination is regarded as a reality of sense, and of course referred to the eye. But it is not necessary even to this result, that the counteracting influence of external sensation or the exercise of judgment should be impaired, for a mere increase in the energy and activity of the imagination may produce pictures of such vividness, as to impose on the consciousness of the individual, in spite of his reason and his external sensations. More commonly, however, the effect is produced by the combined influence of exaltation in the former, and depression in the two latter. Thus a maniac from drink has an excited imagination, and enfeebled organs of sense, in which state he has many strange visions. But the best illustration is found in ghost-seeing. Darkness excites the imagination, while it impairs external vision, under which combination the spectre created by the former is referred, by the consciousness of the individual, to the eye, and made a reality. An application of the whole of what has been here said, may, in a single sentence, be made to the sight-seeing of the mesmeric somniloquist, in whom external sensation, except that of hearing which is limited (we are told) to the person in communication, is suspended, and the reasoning powers are almost torpid. In this condition her attention is aroused by a question, and the imagination, which sometimes nods but never sleeps, supplies the reply, out of visual perceptions, which her dreaming consciousness refers to the eye, and calls it seeing. Indeed, all the answers and remarks of the somniloquist on whom the recent experiments were made, show, that she regarded herself as seeing; but on the theory we are seeking to illustrate, she could no more be conscious that she saw only the works of her own imagination, than the ordinary somnambulist, or the madman from drink, or the superstitious and frightened girl in the darkness of night, is conscious, that what the imagination presents has no existence without.

These views enable us to explain several circumstances, which are said, by those who are familiar with mesmeric exhibitions, to be realities. One is, that some individuals in the somniloquent state have very little mental sympathy. They will either give no intelligible answers, or such as relate chiefly to the body. Now, in the former case, the imagination is no doubt naturally dull, and will not, therefore, embody ideas into intellectually visible forms; but if the phenomena depended on transfusion of ideas from another mind, I can see no reason why they should not appear, as it is easier for water to run into the earth if dry, than wet. In the latter case, when the answers relate chiefly to the body, and the individual is said to have corporeal but not mental sympathy, the case may, I think, be explained in this manner. His existence has been more physical than mental; and his thoughts, his knowledge, and his previous exercises of imagination, have related chiefly, in past times, to the wants and pleasures of the body. Now when such an one, in mesmeric somniloquism, is excited by questions, his imagination does not—cannot I might say—embody ideas, which relate to other objects than those which have administered to his physical enjoyments; and will, therefore, supply him with answers which relate to the body, rather than the mind—by which he will soon become characterized as deficient in mental, but great in bodily, sympathy.

In connexion with this part of our subject, I may refer to what has been so often affirmed, that mesmeric somniloquists seem to be free from all impure thoughts and feelings. This cannot be explained on the hypothesis of transfusion or mental sympathy, but gives support to the opposing theory. The connexion of the imagination with the sentiments and emotions of the soul, is more intimate than with the grosser propensities of the body, and hence, when the chief excitement is in the imagination, the moral feelings are more awakened by its exercises than the animal, and an aspect of mildness, propriety and purity shines forth.

Much has been said on the brilliancy of the intellectual manifestations, which it is alledged are sometimes made by mesmeric somniloquists. I am aware that natural somniloquists have made such displays; and am therefore willing to grant that means of eliciting them from the mesmeric somniloquist may be discovered, as the two states are closely analogous. I must say, however, that in all which the subject of our experiments uttered, nothing of the kind was elicited; but every answer and remark indicated the vacant and languid state of mind, of which I have spoken. But suppose such manifestations should be made, must we necessarily believe them transfused from another mind—mere reflections? Certainly not; seeing that in natural somniloquism, they are the *creations* of the mind of the somniloquist, whose *waking* mental manifestations they often surpass. But if they can come forth as original, not reflected manifestations, from the natural somniloquist, why *may* they not have the same origin in the mesmeric?—Indeed, from the close resemblance of the two, a sound philosophical logic requires us to believe, that the two mental displays have in fact the same origin. But this conclusion excludes the theory of transfusion, which would make the striking displays of the mesmeric somniloquist but the reflected light of a brilliant mind in communication with her own. If this latter were the case, her manifestations should, in their force, bear a relation to the brilliancy of mind of her companion, which seems *not* to be the case; it is affirmed however that they *do* bear a relation to the force and sprightliness of his conversation, indicating that his exciting influence reaches her through the ear. On neither theory can I see room for any extraordinary exhibitions, or a ground for great expectation. On that of transfusion and reflection, nothing can come forth of greater excellence than what is in the transfusing mind, for the amount of light reflected can never exceed that which falls on the reflecting surface; and is not likely to be equal, as some will be absorbed and some refracted. It is a delusion then, if transfusion be true, to expect anything more remarkable than what has been witnessed. On the theory of excitation by the stimulus of questions, I know not that we are warranted in looking for brighter manifestations than have yet been made; but as we cannot foresee to what extent the creative energy of the somniloquist's imagination may be excited by external influence, the theory of interrogative agitation seems to hold out a greater prospect of *original* manifestations than the other; which, indeed, can afford *no* promise whatever of anything original; nor, until the stream can rise higher than the fountain, of anything superior to what is in the transfusing mind.

I cannot refrain from calling attention to the question, how far a mesmeric somniloquist may be educated. We know that in cases of prolonged somniloquism, constituting a kind of double consciousness, in which the paroxysm lasts for several weeks, the subject learns things which in the lucid or sane interval, that follows, are not remembered; but which return in the next somniloquent paroxysm. The same is true of the mesmeric somniloquist on whom our experiments were made. In the presence of several of us, she displayed a personal knowledge of a gentleman who had often been introduced to her, recognized his watch and was fond of handling it. She also recollected her imaginary visit to England with one of our own number, and remembered not only what (it has been assumed) was communicated to her by transfusion, as the seeing of a strange beast; but also what the gentleman said to her in words, as for example, his proposal to visit England and his father's house. Now, if the knowledge acquired in the somniloquent state, or the images and imagery raised in the mind by questionings and conversation, remain with her, (and if she remember from one paroxysm to another, it may be concluded that she remembers much longer,) it follows, that after a time her somniloquent memory will become filled with a great variety; and that she will improve in her conversational powers, especially on certain subjects, those upon which the questions put to her oftenest turn her imagination; and when, at a subsequent time, a question happens to awaken the recollection of an object or group formerly presented to her, she may immediately describe it. In this way, if any one should happen by a question to revive in her mind the falls of Niagara, or the interior of a steam boat, the conception of which had ever been raised by the force of leading questions (to which all somniloquists are known to be amenable) she might instantly give out the scene; and if any one had ever, by interrogation skilfully directed, caused her to imagine, that is, see, a man with one arm only, an inquiry about the arms of a man, might immediately embody to her consciousness that which had long slept. Or, if some one should in a remark of his use the word beast, although he did not intend to present an animal to her view, it might forthwith revive the conception of the strange beast, which she saw in her visit to England. And if an individual, introduced to her a second time, should be recognised and fully remembered by her, his questions might bring up, as recollections, the scenes which had been raised in her mind at the previous interview; although his own might be now in a different state from what it was then. All this is apt to happen in the waking state, as the meeting with an old school fellow revives the memory of events that might not have been recollected for half a century.—Unless she had her ideas associated according to the same laws with our own, we could not have conversed with her—having them so associated, all that I here insist upon seems inevitable.

As further evidence of her being docile, when in the somniloquent state, I may mention her practising with music-teachers and amateurs, before performing with them in public exhibitions; and her telling a lady who had sung with her, that she wished to sing with her again, because she pronounced the words so distinctly. Notwithstanding this evidence that she followed the voice, it is believed by many intelligent persons, that she both sings and reads with others, by mental sympathy. It seems strange, that before coming to a conclusion on this point, the experiment had not been made, of introducing the singer and reader, by their arms being thrust through a partition. If she should sing and read, simultaneously, with them, in such an experiment, when she could not hear their voices, the sympathy of her mind with theirs would be established.

The mode of introducing, or putting a person in communication with the somniloquist, is incompatible with the hypothesis of a material agent, by means of which the transmigration of ideas takes place. Thus the practice at present is, for the mesmerizer to place the hand of the visitor in that of the somniloquist, and then perform on both certain manipulations; still it is not requisite that it should be the hand of the person announced; any other hand will do equally as well. It is only necessary that the somniloquist feel a hand and hear a name, to secure her attention—to make her conscious of the companionship. The hand of one may be grasped by her—the name of another pronounced, when she will immediately answer the question of the latter. This fact has been established as conclusively, both in Cincinnati and Louisville, as any fact can be, and the inference to which it irresistibly leads, is that no physical agent is the efficient cause of the mental intercourse between them, but that it takes place through the ordinary *media* of the external senses and the organs of speech, hearing, and touch. The introduction may even be effected without joining hands, but more easily by it; the reason for which manifestly is, that the attention of the somniloquist is more awakened and specifically directed, when her sense of touch as well as of hearing is appealed to, than when the latter only is addressed. It is well known, moreover, that after having been several times in conversation with a somniloquist, the visitor may introduce himself;—that is, he has become an acquaintance—a familiar—a friend: his voice and touch are perceived, and he is recognized. All this would seem to indicate that the process of social intercourse with the somniloquist is not physical but psychological—not occult but open—not through the vibrations or the transmitting power of a hypothetical, undiscovered etherial fluid; but through the *ordinary media* of air and light, assisted by appropriate impressions on the nerves of feeling. This conclusion concerning the nature of the introduction to a somniloquist, at such utter variance wi h the hypothesis of the transfusion of the thoughts and feelings of another through an unknown channel, is in perfect harmony with the theory of interrogation.

The measures employed, at various periods, to excite the mesmeric state, deserve to be mentioned, as lending no support to the hypothesis of a specific agent, emanating from the Mesmerizer. Those at first employed were entirely different from the present. They were not personal but instrumental. An iron pot, magnets, rods, and ropes with which the subjects were bound together, then constituted the apparatus of a mesmerizer; to these succeeded a complex system of passes with the hand, and other manipulations, all instruments being thrown aside; now, the eye performs, or may perform, the whole; and that too, without anything being radiated from it to the subject, for Faria, one of the oldest and most scientific mesmerizers of Europe, expressly declares, that it is not necessary to make any effort of the will—it is only requisite to make the subject *believe* that such an effort is made, and the somniloquent state occurs. A learned and venerable mesmeric philosopher, of this city, informs me, that he has put a boy into somniloquism, by merely looking at him, when they were ten feet apart. It is quite obvious, that such an effect could only have been produced through the influence of the imagination and feelings.

In conclusion, the variety of aspects which the phenomena of mesmerism have exhibited, deserve to be regarded in connexion with the hypothesis of a specific agent or influence as producing them. The phenomena of the known agencies of nature, such as light, caloric, and electricity, are invariable. Science multiplies them to our observation, but the new do not supersede the old—they are cumulative, and every succeeding year swells the aggregate. Time exerts on the phenomena of mesmerism a very different influence. There is change but no cumulation, progression without aggregation—the snowball rolls on, but melts at the sametime and grows no larger. The soil does not annually bring forth a more abundant harvest as it becomes more thoroughly impregnated with the same seeds, but a new plant supersedes the old, to be in turn displaced by another. The first phenomena of mesmerism, consisted chiefly in various agitations of the muscular system—in spasms, hysteria, syncope, coughs and vomiting; to these succeeded somniloquism, with a vision so quickened, that the individual could see deeper into a mill-stone than he who picks it:—a *clairvoyance* of the eye, which could discover what was then transacting in distant places; a *clairvoyance* of the mind, that enabled the somniloquist to penetrate the arcana of science; a *prevoyance* that could perceive the shadows of coming events, when other eyes could descry nothing! But this brilliant coruscation on the face of humanity, like a meteor of the heavens, soon passed away,—and is now succeeded by the phenomena of metempsychosis. From a state of beatific inspiration, the devoted somniloquist is degraded to the condition of a mere passive and unresisting recipient of the thoughts, feelings and will of those in communication. Her ideas are no longer her own;—she is compelled to feel what others feel;—she cannot move but at their bidding. The barriers of her mind are broken down, and "blue spirits and black, white spirits and gray," enter without opposition, and revel in its mansions without molestation. Her personal consciousness has become a *tertium quid*, composed of her own and that of another united. She is transformed into a spiritual hybrid, and loses her accountability both to God and man, as the laws of neither recognize such personality.

Louisville, Feb. 10, 1844.

